# Characterization of Antibiofilm Molecules from Bovine Coagulase-Negative Staphylococci

**DOI:** 10.1371/journal.pone.0351675

**Published:** 2026-06-30

**Authors:** Coralie Goetz, Ibtissem Doghri, Sneha Das, Audrey Corbeil, Pauline Bouveret, Francis Beaudry, David Lalaouna, Eric Massé, Mario Jacques, François Malouin

**Affiliations:** 1 Département de pathologie et microbiologie, Faculté de médecine vétérinaire, Université de Montréal, St-Hyacinthe, Québec, Canada; 2 Regroupement de recherche pour un lait de qualité optimale (Op+lait), St-Hyacinthe, Québec, Canada; 3 Département de biomédecine vétérinaire, Faculté de médecine vétérinaire, Université de Montréal, St-Hyacinthe, Québec, Canada; 4 Centre de recherche sur le cerveau et l’apprentissage (CIRCA), Montréal, Québec, Canada; 5 Département de biochimie et génomique fonctionnelle, Université de Sherbrooke, Sherbrooke, Québec, Canada; 6 Département de biologie, Faculté des sciences, Université de Sherbrooke, Sherbrooke, Québec, Canada; Universidad Autonoma de Chihuahua, MEXICO

## Abstract

Bacterial biofilms are structured communities of bacterial cells enclosed in a self-produced polymeric matrix, which can adhere to biotic or abiotic surfaces. This mode of existence permits these bacteria to endure in adverse conditions, including the presence of antibiotics Bacteria within biofilms are responsible for numerous infections in humans and animals, including bovine mastitis. It is therefore important to develop new therapeutic strategies to control and treat biofilm-associated infections. The results obtained by our group during the study of mixed bacterial biofilm communities showed that four isolates of coagulase-negative staphylococci (CNS; two *Staphylococcus chromogenes* and two *Staphylococcus simulans*) that produce only a small amount of biofilm can significantly reduce biofilm formation in approximatively 80% of pathogenic staphylococci associated with bovine mastitis. Furthermore, supernatants of *S. chromogenes* reduced secondary intramammary colonization by *S. aureus* in a murine model of mastitis. However, information regarding the mechanism and the effector molecule(s) involved is lacking. The objective of this study was therefore to investigate and characterize the antibiofilm molecule(s) produced by these four CNS isolates. In this context, we prepared culture supernatants from two isolates of *S. chromogenes* (C and E) and two isolates of *S. simulans* (F and H) to evaluate their effect on biofilm production of pathogenic bacterial species involved in bovine mastitis. Using a standard biofilm microtiter plate assay, we demonstrated that the four CNS supernatants not only have a significant impact on biofilms of pathogenic staphylococci (68% of tested isolates) but also on those of other important mastitis pathogens such as *Streptococcus* spp., *Trueperella pyogenes*, *Klebsiella* spp. and *Escherichia coli* (61.3% of tested isolates)*.* The isolation and characterization of the antibiofilm molecule(s) contained in the supernatants were then conducted using a filtration process with membranes of different porosities, as well as through physicochemical and enzymatic treatments. We were then able to confirm that antibiofilm activity against staphylococci was present in the < 3kDa fractions of CNS culture supernatants and that this activity was heat-stable and protease-resistant, but sensitive to RNase A, suggesting that the antibiofilm activity might be due, at least in part, to an RNA molecule. Preliminary results showed that the antibiofilm activity was maintained with RNA extracts from fractioned supernatants (<3kDa). In conclusion, these results confirmed that some CNS have an antibiofilm activity, which represents a promising new avenue in the fight against biofilm-associated infections, particularly bovine mastitis.

## Introduction

Coagulase-negative staphylococci (CNS) have traditionally been considered commensals of humans and animals; however, some species are now recognized as etiologic agents of infections such as healthcare-associated infections in humans and mastitis in animals [1–5]. The ability of these pathogens to persist in host tissues for extended periods is extremely problematic. The evasion of host immune defenses and antibiotic treatment by these bacteria involves multiple mechanisms, including biofilm formation [6–8]. Biofilm formation is a common bacterial trait in which cells are enclosed within a matrix they produce themselves and attached to surfaces [2,9]. Furthermore, large bacterial aggregates – which do not adhere to a surface but exhibit many of the same phenotypes as surface-attached biofilms, including increased antibiotic resistance/tolerance – are also frequently observed in both clinical (e.g., including bovine mastitis) and environmental contexts [10–13]. In *Staphylococcus aureus*, biofilm formation is associated with persistent infections such as bovine mastitis as well as a loss of susceptibility to antibiotic treatments [3,14]. For CNS, biofilm formation also appears to contribute to persistence in the mammary gland, as it has been associated with reduced susceptibility to biocides and antibiotics, and a strong correlation between biofilm thickness and the number of days of lactation has been observed [15,16]. Several hypotheses explain the reduced susceptibility of bacteria present in biofilms to antimicrobial agents [9,17,18]. First, the polymer matrix acts as a barrier and reduces, or even prevents, the diffusion of the antibiotic into the biofilm. Thereby, the antibiotic concentration will not be sufficient to neutralize the targeted bacteria. Second, the polymer matrix has electrostatic charges on its surface that bind to certain antimicrobial agents. Again, this phenomenon prevents an effective concentration of antibiotic from being reached within the biofilm matrix. Finally, another explanation concerns the state of the bacterial cells inside the biofilm, which, due to low levels of nutrients and oxygen, will be in a dormant state and will therefore not be effectively affected by the antibiotics. These persistent cells will then allow the biofilm, weakened by antibiotic treatment, to repopulate and lead to a chronic infection [19]. Furthermore, bacteria present in a biofilm composed of multiple species (mixed biofilm) exhibit reduced sensitivity to antibiotics compared to bacteria present in a biofilm composed of a single species [17]. Consequently, the resistance or tolerance of bacteria within biofilms could be responsible for the failure of certain antibiotic therapies [20], which motivates the development of new antibiofilm strategies, whether or not in combination with antibiotic treatment [21,22].

The results obtained by our group in the study of biofilms composed of mixed bacterial species showed that four CNS isolates having a weak biofilm-forming phenotype, two *Staphylococcus chromogenes* and two *Staphylococcus simulans*, can significantly reduce biofilm formation in approximately 80% of the tested staphylococcal species, including *Staphylococcus aureus* [4]. They were also capable of dispersing a pre-established biofilm, albeit to a lesser extent [4]. More recent results obtained by our team have shown that *S. chromogenes* supernatants were capable of reducing secondary intramammary colonization by *S. aureus* [23]. However, the mechanisms behind the inhibition of biofilm formation and its dispersion (*i.e.*, biofilm detachment) have not yet been characterized, and their effect on other important Gram-positive and Gram-negative mastitis pathogens, including *Streptococcus* spp., *Trueperella pyogenes*, *Klebsiella* spp., and *Escherichia coli* should also be evaluated to determine the spectrum of this activity.

The objective of this study was therefore to investigate and characterize the antibiofilm molecules produced by four isolates of CNS that act against various pathogens isolated in cases of bovine mastitis.

## Materials and methods

### Bacterial isolates and growth conditions

The isolates were obtained from the Mastitis Pathogen Culture Collection (MPCC), managed by Op+lait (oplait.org, St-Hyacinthe, Québec, Canada; [24,25]). The bacterial isolates used in this study are listed in Table 1. Coagulase-negative staphylococci, *Staphylococcus aureus*, *Escherichia coli*, *Klebsiella* spp*.* and *Streptococcus* spp. were routinely cultured on brain heart infusion (BHI) agar and incubated for 24h at 37°C, with the exception of *E. coli*, which was incubated at 30°C. *Trueperella pyogenes* was cultured on BHI agar and incubated for 48h at 37°C with 5% CO_2._

**Table 1:** Biofilm formation conditions.

Bacterial Isolates	Growth Conditions	Corning Plate ID	Incubation conditions	Incubation time (h)	Staining	References
*S. chromogenes* (n = 7; #101-#105 and C and E)^2^ *S. simulans* (n = 7; #106-#110 and F and H) *S. xylosus* (n = 5; #111-#115) *S. epidermidis* (n = 5; #116-#120) *S. haemolyticus* (n = 5; #121-#125) *S. aureus* (n = 5; #126-#130)	Colonies from BHI agar were suspended in BHI + glucose ^1^ to a 0.5 McFarland standard	3595 3516	37°C	24	0.1% (w/v) safranin for 10 min	[4,15]
*S. uberis* (n = 5; #131-#135) *S. dysgalactiae* (n=5; #136-#140)	Colonies from BHI agar were suspended in BHI + glucose to a 0.5 McFarland standard	3595	37°C	24	0.1% (w/v) safranin for 10 min	This study
*T. pyogenes* (n = 5; #141-#145)	Colonies from BHI agar were suspended in BHI + glucose to a 0.5 McFarland standard	3595	37°C, 5%CO_2_	48	0.1% (w/v) crystal violet for 10 min	This study
*E. coli* (n = 5; #146-#150)	Colonies from LB agar were cultured O/N at 30°C with shaking in M9G supplemented with glucose (M9G; 0.25% w/v) and this culture was diluted (1/100) in fresh M9G.	3370	30°C	24	0.1% (w/v) crystal violet for 10 min	[26]
*K. oxytoca* (n = 5; #151-#156) *K. pneumoniae* (n = 5; #157-#160)	Colonies from BHI agar were suspended in BHI + glucose to a 0.5 McFarland standard	3595	37°C	24	0.1% (w/v) crystal violet for 10 min	This study

^1^BHI supplemented with glucose (0.25% w/v)

^2^Numbers and letters in parenthesis represent the strain numbers shown in the Tables and Figures of this work.

### Preparation of CNS culture supernatants

*Staphylococcus chromogenes* isolates C and E, as well as *Staphylococcus simulans* isolates F and H, which produce only a small amount of biofilm, were cultured in BHI medium supplemented with glucose (0.25% w/v; BHI + glucose) in 6-well microtiter plates (Corning Costar #3516, Corning, NY, USA), as previously described [4]. Briefly, colonies from BHI agar were resuspended in BHI + glucose to a 0.5 McFarland standard and 9 mL were placed in each well. The plate was then incubated without agitation for 24 h at 37°C to promote biofilm formation. After incubation, the supernatant was collected, centrifuged at 4,000 rpm (2,800 g) for 20 min at 4ºC, sterilized by filtration (0.2 µm, Sarstedt Filtropur S, Nümbrecht, Germany) [27], and then stored at −80°C until use.

### Biofilm assays

#### Microtiter plate assay.

**Fig 1:**
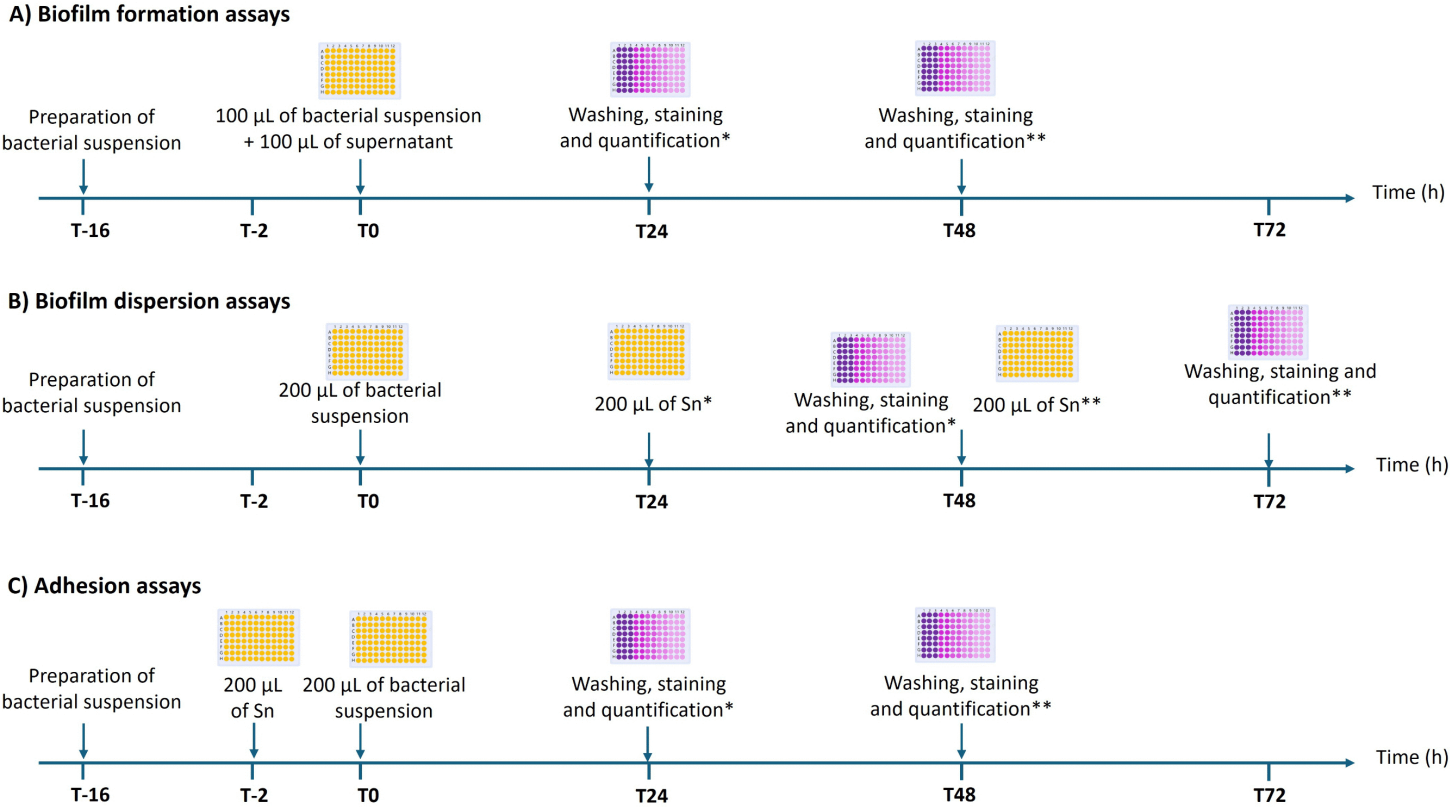
Experimental design of biofilm quantification using staining techniques. **Microtiter plate assays followed by biofilm staining were used to evaluate the effect of CNS supernatant to inhibit biofilm formation (A), to induce biofilm dispersion (B) or to inhibit initial cell adhesion (C).** Abbreviations: Sn, supernatant; h, hour.

**Quantification by staining techniques.** For biofilm formation assays (Fig 1), bacterial suspensions of *Staphylococcus* spp*.*, *Streptococcus* spp*.*, *T. pyogenes*, *E. coli* and *Klebsiella* spp. were cultured in the appropriate growth medium (Table 1). To investigate antibiofilm activity, 100 μL of bacterial suspension were loaded with 100 μL of CNS supernatant in a 96-well microtiter plate, for a total volume of 200 μL. After 24 or 48 h of incubation (Table 1), planktonic cells were removed, and the wells were washed three times with PBS. The biofilms present in the wells were air-dried and stained with 0.1% (w/v) safranin for 10 min [4,15] or with 0.1% (w/v) crystal violet for 10 min [26], depending on the species (Table 1). The stain was then dissolved in 200 μL of the destaining solution [50% (v/v) ethanol, 50% (v/v) glacial acetic acid] and quantified by measuring the absorbance at 490 nm (A490). At the same time, the effect of the CNS supernatants on bacterial growth was assessed by measuring the absorbance at 600 nm (A600). In each experiment, single-species biofilms complemented with sterile BHI + glucose instead of the CNS supernatant acted as controls. Furthermore, 200 µL of sterile BHI + glucose were added to the wells to act as blank wells.

For biofilm dispersion assays (Fig 1), single-species biofilms were cultured as described in Table 1. After 24 or 48 h of incubation, the planktonic cells were removed, and then 200 µL of CNS supernatant were added to the wells (Table 1). For the control, biofilms were further incubated with 200 µL of BHI + glucose without the addition of CNS supernatant. After 24 or 48 h, the biofilms were stained and quantified as described above.

For adhesion assays (Fig 1), single-species biofilms were cultured as previously described following a 2-h pre-incubation of the wells with 200 µL of each supernatant. For the control, the wells were incubated further with 200 µL of BHI + glucose without the addition of CNS supernatant.

Each assay was repeated on three independent days (i.e., three biological replicates), with three technical replicates performed each day [4,15]. The plate layout was modified at each assay to avoid a positional bias.

The effects of the supernatants on biofilm formation, dispersion or adhesion were calculated based on the mean of 3 biological replicates with the following formula = (100%) – (% of remaining biofilm relative to control), where 100% is the amount of biofilm formed in BHI+glucose, and were expressed as a percentage (%) of the remaining biofilm relative to a control biofilm without CNS supernatant.

**Confocal laser scanning microscopy.** Biofilms, with or without treatment with CNS supernatant, were prepared as described above in 96-well microtiter plates, then stained using the SYTO® 9 green fluorescent nucleic acid stain (Invitrogen, Waltham, MA, USA) or with FilmTracer^TM^ FM 1–43® fluorescent marker (Molecular Probes; Eugene, OR, USA), as recommended by the manufacturers. The stained biofilms were visualized by confocal laser scanning microscopy (CLSM; FV1000 IX81; Olympus, Markham, ON, Canada) and images were acquired using Fluoview software (Olympus). The biofilm biovolume (BV) was quantified using Image pro software (Media Cybernetics, Silver Spring, MD, USA).

### Microfluidic assay

Dynamic biofilm growth was assessed using the BioFlux 200 device (Fluxion Biosciences, South San Francisco, CA, USA), as described previously [4]. Briefly, bacteria were suspended in 4 mL of prewarmed (37°C) BHI + glucose medium until an optical density at 600 nm of approximately 1 was achieved. The microfluidic channels were wetted with prewarmed BHI + glucose, then inoculated by injecting the bacterial suspension into the output reservoir for 20 s at 0.5 dyne/cm^2^. The microfluidic plate was incubated for 1 h at 37°C to allow the bacteria to adhere to the surface. Fresh prewarmed BHI + glucose medium or CNS supernatant were added to the input reservoir, and the flow of these media was initiated at 0.5 dyne/cm^2^ for 23 h. Next, the biofilms were washed by injecting phosphate-buffered saline (PBS) from the input reservoir for 20 min at 0.5 dyne/cm^2^. Images of the biofilms were obtained using a microscope equipped with a 40 × objective (CKX41; Olympus), a digital camera (Retiga EX; QImaging, Surrey, BC, Canada) and the software supplied with the BioFlux 200 device.

### Physicochemical analysis and characterization of CNS active compounds

Various treatments were applied to the CNS supernatants to determine the nature of their antibiofilm activity. The CNS supernatants were first fractionated using Microcon centrifugal concentrators (Millipore, Billerica, Massachusets, USA) equipped with filters having cut-off sizes of 3- and 10-kDa. To assess the heat sensitivity of the active compounds, the fractioned CNS supernatants (<3kDa) were treated for 15 min at 100°C [27]. To assess the chemical polarity of the active compounds, the fractioned CNS supernatants (<3kDa) were separated using a ZEOprep 60 C_18_ reversed phase column (Zeochem, Rüti, Switzerland). To characterize the nature of the active compounds, the fractioned supernatants (<3kDa) were treated with various enzymes or NaOH. They were incubated 4 h at 37°C with proteinase K (Sigma, St. Louis, Missouri, USA) (1 mg/mL) [28]; 12 h at 37°C with RNase A (Sigma) (25 μg/mL) or DNase I (Sigma) (100 μg/mL) [29]; 48 h at 37°C with lipase (Sigma) (2 mg/mL) [29]; 30 min at 30°C with dispersin B (Kane Biotech, Winnipeg, MB, Canada) (50 μg/mL) [30]; 30 min at 37°C with α-amylase (Sigma) (1 mg/mL) [31] or 1 h at 37°C with lysostaphin (Sigma) (0.1 mg/mL) [32]. After incubation, the enzymes were removed using filters with cut-off sizes of 3, 10 or 50-kDa (Millipore, Billerica, Massachusetts, USA). NaOH was also used to alkalinize (to a pH of approximatively 11) the CNS fractions for 1 h at room temperature [33].

All treated samples, along with their respective controls, were then tested for antibiofilm activity using the biofilm formation assays described above. Each assay was repeated on three independent days, with three technical replicates performed each day.

### RNA extraction and sequencing

RNA molecules were extracted from CNS supernatants using a phenol-based method adapted from [34]. Briefly, a DNase treatment was performed on the fractioned supernatants (<3kDa), followed by extraction using a 25:24:1 (v/v) mixture of phenol, chloroform and isoamyl alcohol. The RNA was then precipitated by adding 3 volumes of ethanol to the aqueous phase, followed by overnight incubation at −80°C. The RNA precipitate was collected by centrifugation and resuspended in 30 µL of distilled water. After RNA purification, cDNA libraries were prepared using the ScriptSeq v2 RNA-Seq Library Preparation Kit (Epicenter). The samples were then sequenced on a MiSeq sequencing system (Illumina) from the RNA platform at the Université de Sherbrooke (https://rnomics.med.usherbrooke.ca/).

The Galaxy Project and UCSC Microbial Genome Browser [35,36] were used to analyze and visualize the sequencing data,. FASTQ Groomer was used to verify and convert FASTQ files [37]. The quality of the raw sequencing data was assessed using FastQC:Read QC. Next, the reads were aligned to the *Staphylococcus* spp. genome assembly using Map with Bowtie for Illumina (version 1.1.2). Create a BedGraph of genome coverage (version 0.1.0) was used to visualize reads *Staphylococcus* spp. genome with UCSC Microbial Genome Browser.

### Statistical analysis

Each assay was repeated on three independent days (i.e., three biological replicates), with three technical replicates performed each day. For the microtiter plate assays, the data were analyzed using one-way ANOVA after verifying normal distribution (Shapiro-Wilk test), and Dunnett’s test was used to compare the mean of 3 biological replicates with control samples (GraphPad Prism Version 5.03 software). Statistical significance is established for p-values less than 0.05 (p < 0.05). The results are reported to two or three decimal places.

## Results

### Antibiofilm activity is found in *S. chromogenes* and *S. simulans* culture supernatants

**Fig 2:**
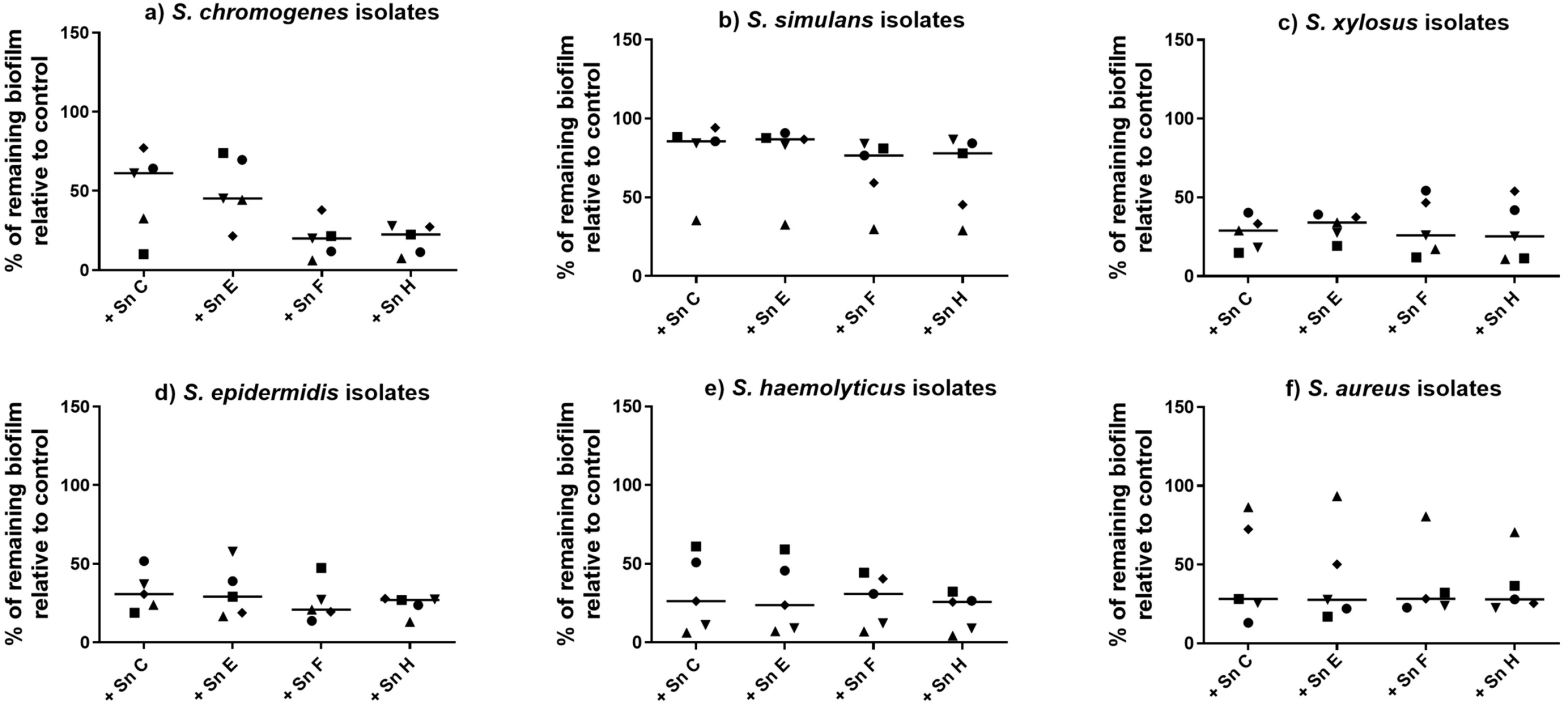
Activity of the supernatants from *S. chromogenes* and *S. simulans* on biofilm formation in staphylococcal isolates exhibiting a strong biofilm phenotype. Activity of 4 supernatants of *S. chromogenes* (Sn C and Sn E) and *S. simulans* (Sn F and Sn H) isolates exhibiting a weak biofilm phenotype (x axis) on biofilm formation in five isolates exhibiting a strong biofilm phenotype representing six species of staphylococcal: *S. chromogenes* **(a)**, *S. simulans* **(b)**, *S. xylosus* **(c)**, *S. epidermidis* **(d)**, *S. haemolyticus* (e) and *S. aureus* **(f)**. The effects of the supernatants on biofilm formation were calculated with the following formula = (100%) – (% of remaining biofilm relative to control), where 100% is the amount of biofilm formed in BHI+glucose, and were expressed as a percentage (%) of the remaining biofilm relative to a control biofilm without CNS supernatant. Each dot represents the mean of triplicates obtained over three independent days for each isolate. The 5 different symbols represent the 5 different isolates exhibiting a strong biofilm phenotype for each species (see S1 Table). The bars represent the median values for each group. Statistical differences are presented in S1 Table. Abbreviations: Sn, supernatant.

**Fig 3:**
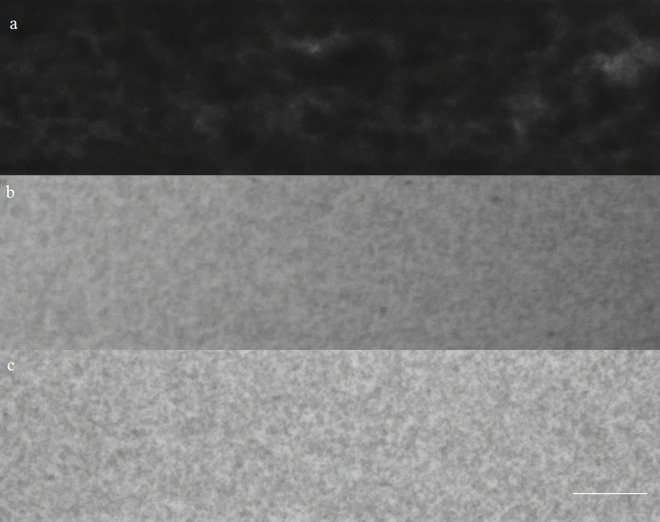
Activity of the supernatants from *S. chromogenes* and *S. simulans* on biofilm formation in a staphylococcal isolate exhibiting a strong biofilm phenotype, using a microfluidic system. Microscopic images of biofilms formed by a CNS isolate with a strong biofilm phenotype (*S. simulans* #108) cultured with a control broth (BHI, a) or with supernatants from *S. chromogenes* C (b) or *S. simulans* F **(c)**. The images were obtained after 24 h of culture in a flow chamber of the BioFlux 200 microfluidic system using a microscope equipped with a 40 × objective, a digital camera and the software supplied with the BioFlux 200 device. Scale bar = 100 µm.

Our previous study, conducted on mixed biofilms, showed that four CNS isolates with a weak-biofilm phenotype (*S. chromogenes* C and E, as well as *S. simulans* F and H) could inhibit biofilm formation in other staphylococci associated with bovine mastitis [4]. *S. chromogenes* supernatants also prevented secondary intramammary colonization by *S. aureus* [23]. We therefore decided to investigate the ability of culture supernatants from CNS isolates that impact biofilm formation in staphylococci isolates exhibiting a strong biofilm phenotype. Biofilm assays revealed different activity profiles depending on the CNS species and isolates, as well as on the CNS supernatant (Fig 2, S1 Table). Biofilm formation in all isolates of *S. chromogenes, S. xylosus, S. epidermidis,* and *S. haemolyticus* that exhibited a strong ability to produce biofilm was significantly reduced (p < 0.05; a reduction ranging from 49.1% to 80.7% compared to biofilm treated with sterile BHI + glucose) by the four supernatants (Fig 2a, 2c, 2d and 2e, S1 Table). A significant reduction (p < 0.05, ranging from 54.8% to 63.4%) in biofilm formation was also observed for certain *S. aureus* isolates exhibiting a strong biofilm phenotype when cultured in the presence of CNS supernatants (Fig 2f, S1 Table). We observed a more pronounced effect of *S. simulans* supernatants on the inhibition of biofilm formation by *S. chromogenes* (80.63 vs 50% of inhibition; Fig 2a). With the exception of one isolate (#108, S1 Table), biofilm formation by *S. simulans* was not affected by the supernatants tested (Fig 2b). The antibiofilm activity previously observed in mixed biofilms [4] is thus present in cell-free CNS culture supernatants. These results were subsequently confirmed under dynamic conditions using a microfluidic system. For example, *S. simulans* #108, a strong biofilm producer, was unable to form its typical robust biofilm (Fig 3a) in the presence of supernatants C (Fig 3b) or F (Fig 3c) derived from *S. chromogenes* or *S. simulans* respectively, after a 24 h incubation (Fig 3).

### Dispersion of established biofilms by *S. chromogenes* and *S. simulans* supernatants

**Fig 4:**
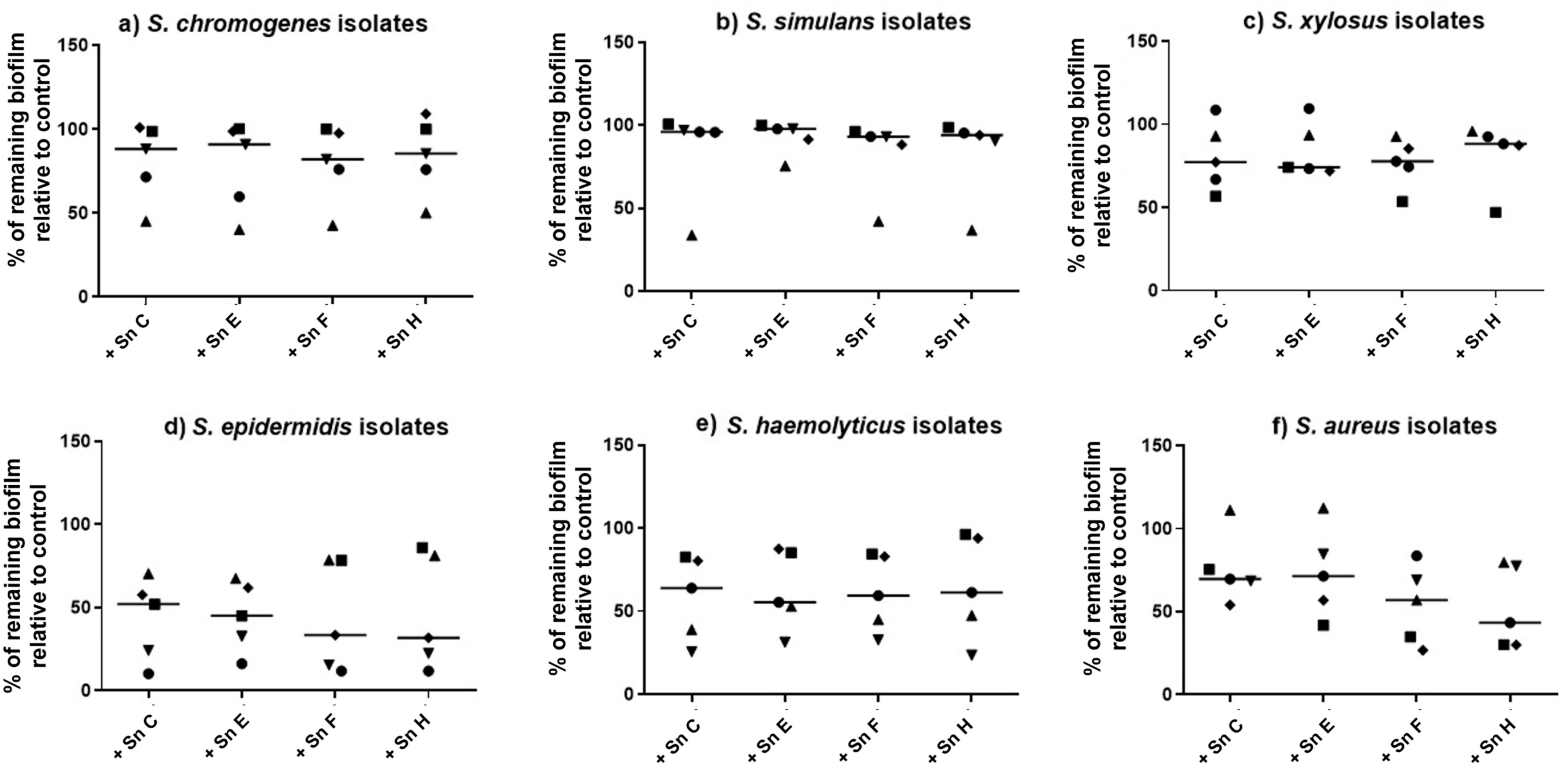
Activity of the supernatants of *S. chromogenes* and *S. simulans* on pre-established biofilms derived from staphylococcal isolates exhibiting a strong biofilm phenotype. Activity of 4 supernatants from isolates of *S. chromogenes* (Sn C and Sn E) and *S. simulans* (Sn F and Sn H) exhibiting a weak biofilm phenotype (x axis) on pre-established biofilms derived from five isolates exhibiting a strong biofilm phenotype representing six species of staphylococci: *S. chromogenes* **(a)**, *S. simulans* **(b)**, *S. xylosus* **(c)**, *S. epidermidis* **(d)**, *S. haemolyticus* (e) and *S. aureus* **(f)**. The effects of the supernatants on pre-established biofilms were calculated with the following formula = (100%) – (% of remaining biofilm relative to control), where 100% is the amount of biofilm formed in BHI+glucose, and were expressed as a percentage (%) of the remaining biofilm relative to a control biofilm without CNS supernatant. Each dot represents the mean of the triplicates obtained over three independent days for each isolate. The 5 different symbols represent the 5 different isolates of each species (see S2 Table). The bars represent the median values for each group. Statistical differences are presented in S2 Table. Abbreviation: Sn, supernatant.

Our results showed that the four CNS supernatants can reduce the formation of staphylococcal biofilms (previous section). We therefore also investigated the ability of these CNS supernatants to disperse pre-established staphylococcal biofilms through the active detachment of bacterial cells. Overall, we observed a weaker effect of CNS supernatants on biofilm dispersion compared to that observed on biofilm formation (Fig 4 and S2 Table). It should be noted, however, that the integrity of pre-established biofilms by *S. epidermidis* isolates was most sensitive to CNS supernatants (reduction ranging from 42.89% to 46.96%), particularly to *S. chromogenes* supernatants (p < 0.05; Fig 4d, S2 Table). Furthermore, all 4 CNS supernatants have the ability to significantly disperse pre-established biofilms of *S. chromogenes* (3/5 isolates), *S. xylosus* (3/5) and *S. haemolyticus* (3/5) (Fig 4c, 4e and 4f and S2 Table), whereas pre-established biofilms of *S. simulans* (1/5) and *S. aureus* (1/5) were generally unaffected by the CNS supernatants (Fig 4a and 4b). These dispersion results are consistent with our previous observations on mixed biofilms [21].

### Spectrum of activity of the *S. chromogenes* and *S. simulans* supernatants

**Fig 5:**
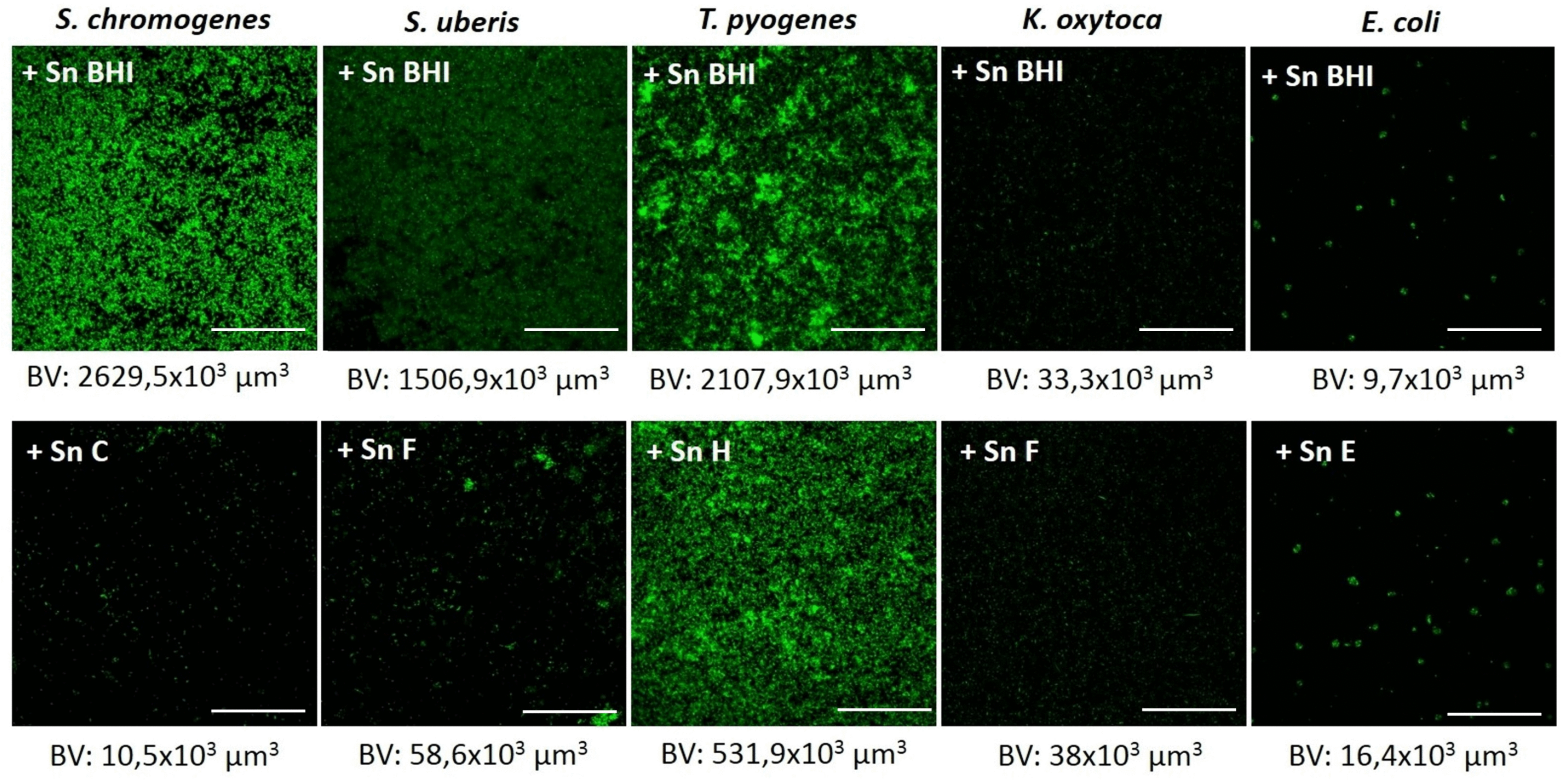
Confocal laser scanning microscopy images of biofilm formation by various mastitis pathogens. Visualization by laser scanning confocal microscopy of biofilm formation by isolates exhibiting a strong biofilm phenotype (*S. chromogenes* #104; *S. uberis* #131; *T. pyogenes* #143; *K. oxytoca* #156; *E. coli* #149) with the addition of control broth (Sn BHI) or one of the CNS supernatants (Sn C, Sn F, Sn H, Sn F or Sn E, respectively). Biovolume (BV) is expressed in µm^3^. A reduction in the BV of biofilms of *S. chromogenes*, *S. uberis* and *T. pyogenes* was observed after the addition of Sn C, Sn F or Sn H, respectively. Abbreviations: Sn, supernatant; BHI, Brain Heart Infusion. Scale bar = 100 µm.

Having observed that the four CNS supernatants could inhibit biofilm formation in various species of staphylococci, we decided to study their effect on other important pathogens responsible for mastitis, including both Gram-positive (*Streptococcus* spp. and *T. pyogenes)* and Gram-negative (*Klebsiella* spp., and *E. coli)* bacteria. The spectrum of activity of the four CNS supernatants on biofilm formation and dispersion was also analyzed using microtiter biofilm assays (Tables 2 and 3, respectively). In general, we observed that the four supernatants significantly inhibited (p < 0.05) biofilm formation in most Gram-positive isolates (ranging from 12/15–13/15 isolates, depending on the supernatant) (Table 2). These observations were confirmed by visualizing with a confocal microscope the biofilm formation in one isolate from each genus (*S. chromogenes* #104; *S. uberis* #131; *T. pyogenes* #143; *E. coli* #149; *K. oxytoca* #156) cultured in the presence of one of the four CNS supernatants (Fig 5). Furthermore, CNS supernatants can significantly (p < 0.05) disperse pre-established Gram-positive biofilms (in 10/15–14/15 isolates) as well as Gram-negative biofilms (from 6/15–13/15 isolates) (Table 3). Overall, antibiofilm activity of CNS is not limited to other staphylococci, and the supernatants of *S. simulans* exhibited a broader spectrum of activity than those of *S. chromogenes* (Tables 2 and 3).

**Table 2:** Proportion of Gram-positive and Gram-negative isolates in which biofilm formation is significantly inhibited by supernatant of *S. chromogenes* (C or E) or *S. simulans* (F or H) as indicated.

	*S. chromogenes*		*S. simulans*	
	C	E	F	H
***S. uberis***	5/5^1^	5/5	5/5	5/5
***S. dysgalactiae***	3/5	3/5	3/5	3/5
***T. pyogenes***	4/5	5/5	5/5	5/5
***E. coli***	0/5	0/5	0/5	0/5
***K. oxytoca***	0/5	0/5	0/5	0/5
***K. pneumoniae***	2/5	2/5	2/5	2/5

^1^n/N, where n is the number of isolates significantly inhibited by the indicated supernatant (p < 0.05) and N the number of isolates tested. For each isolate, the percentage of the remaining biofilm relative to a control biofilm without CNS supernatant was calculated based on the mean of 3 replicates. Statistical significance for the effect on biofilm formation was established for p-values less than 0.05 (p < 0.05).

**Table 3:** Proportion of Gram-positive and Gram-negative isolates in which biofilm is significantly dispersed by the supernatant of *S. chromogenes* (C or E) or *S. simulans* (F or H) as indicated.

	*S. chromogenes*		*S. simulans*	
	C	E	F	H
***S. uberis***	4/5^1^	4/5	3/5	3/5
***S. dysgalactiae***	5/5	5/5	5/5	3/5
***T. pyogenes***	5/5	4/5	4/5	4/5
***E. coli***	4/5	4/5	4/5	4/5
***K. oxytoca***	1/5	2/5	5/5	5/5
***K. pneumoniae***	1/5	1/5	4/5	4/5

^1^n/N, where n is the number of isolates significantly inhibited by the indicated supernatant (p < 0.05) and N the number of isolates tested. For each isolate, the percentage of the remaining biofilm relative to a control biofilm without CNS supernatant was calculated based on the mean of 3 replicates. Statistical significance for the effect on biofilm was established for p-values less than 0.05 (p < 0.05).

### Physicochemical properties and characterization of CNS active compounds

**Fig 6:**
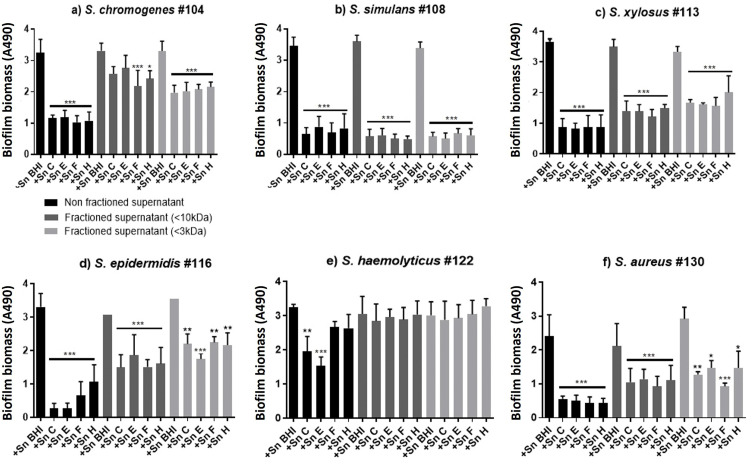
Activity of fractioned supernatants from *S. chromogenes* and *S. simulans* on biofilm formation in staphylococcal isolates exhibiting a strong biofilm phenotype. Inhibition of biofilm formation by non-fractioned supernatants (black) and fractioned supernatants (<10kDa: dark grey; < 3kDa: light grey) (x axis) against an isolate exhibiting a strong biofilm phenotype, representing six species of staphylococci: *S. chromogenes* **(a)**, *S. simulans* **(b)**, *S. xylosus* **(c)**, *S. epidermidis* **(d)**, *S. haemolyticus* (e) and *S. aureus* **(f)**. Biofilm formation is expressed by measurements at 490 nm. A one-way ANOVA analysis was used to obtain the statistical data. * p-value less than 0.05 (p < 0.05); ** p < 0.01; *** p < 0.001. Abbreviations: Sn, supernatant; BHI, Brain Heart Infusion.

To determine the nature and characteristics of the molecules involved in antibiofilm activity, we first fractionated the four supernatants using 10- and 3-kDa cut-off filters (Fig 6). The different fractions were tested against a representative isolate of each staphylococcal species. The results showed that antibiofilm activity was mainly retained in the < 10kDa and <3kDa fractions (Fig 6a-d, 6f). Biofilm formation by *S. haemolyticus* was not affected by the < 10kDa and <3kDa fractions (Fig 5e). We then subjected the < 3kDa fractions, which are the most purified fractions that remained active (Tables 4 and 5), to various treatments (heat treatment, solid-phase extraction and enzymatic treatments) and evaluated their activities against the 5 susceptible staphylococcal isolates (*S. chromogenes*, *S. simulans*, *S. xylosus*, *S. epidermidis,* and *S. aureus*). The active compounds in the CNS supernatant appear to be heat-stable and hydrophilic (p < 0.05; Table 4). We also demonstrated that the activity of the supernatants was not significantly affected by proteinase K, DNase I, lipase, lysostaphin, α-amylase or NaOH (p < 0.05; Table 5). However, the active fractions became less effective against the *S. aureus* isolates after treatment with dispersin B, and more strikingly, the antibiofilm activity was partially or completely lost after treatment with RNase A (Table 5). The results of RNA quantification and sequencing indicate the presence of small RNAs (sRNAs; S1 and S2 Figs and S3 and S4 Tables). Indeed, the electropherogram obtained after RNA extraction from supernatants C, E, F and H revealed the presence of sRNAs (approximatively 25 nucleotides in length). Furthermore, preliminary results showed that RNA extracted from the fractioned CNS supernatants (SN PCl) led to a significant inhibition of biofilm formation by an isolate of *S. chromogenes* (S3 Figs).

**Table 4:** Proportion of CNS isolates and *S. aureus* isolates in which biofilm formation is significantly inhibited by the supernatant (fraction < 3kDa) of the indicated isolate of *S. chromogenes* (C or E) or *S. simulans* (F or H) following the specified treatment.

		*S. chromogenes*		*S. simulans*	
		C	E	F	H
**Heat treatment** **(100°C, 15 min)**	CNS *S. aureus*	4/4^1^ 1/1	4/4 1/1	4/4 1/1	4/4 1/1
**SPE** ^**2**^ **(Aqueous phase)**	CNS *S. aureus*	4/4 1/1	4/4 1/1	4/4 1/1	4/4 1/1
**SPE** **(Hydrophobic phase)**	CNS *S. aureus*	0/4 0/1	0/4 0/1	0/4 0/1	0/4 0/1

^1^n/N, where n is the number of isolates significantly inhibited by the indicated supernatant (p < 0.05) and N the number of isolates tested. For each isolate, the percentage of the remaining biofilm relative to a control biofilm without CNS supernatant was calculated based on the mean of 3 replicates. Statistical significance for the effect on biofilm formation was established for p-values less than 0.05 (p < 0.05). ^2^ SPE: solid-phase extraction.

**Table 5:** Proportion of CNS isolates and *S. aureus* isolates in which biofilm formation is significantly inhibited by the supernatant (fraction < 3kDa) of the isolates of *S. chromogenes* (C or E) or *S. simulans* (F or H) following the specified treatment.

		*S. chromogenes*		*S. simulans*	
		**C**	**E**	**F**	**H**
**Proteinase K**	CNS *S. aureus*	4/4^1^ 1/1	4/4 1/1	4/4 1/1	4/4 1/1
**RNase**	CNS *S. aureus*	2/4 0/1	2/4 0/1	2/4 0/1	2/4 0/1
**DNase**	CNS *S. aureus*	4/4 1/1	4/4 1/1	4/4 1/1	4/4 1/1
**Lipase**	CNS *S. aureus*	4/4 1/1	4/4 1/1	4/4 1/1	4/4 1/1
**Dispersin B**	CNS *S. aureus*	4/4 0/1	4/4 0/1	4/4 0/1	4/4 0/1
**α-amylase**	CNS *S. aureus*	4/4 1/1	4/4 1/1	4/4 1/1	4/4 1/1
**Lysostaphin**	CNS *S. aureus*	4/4 1/1	4/4 1/1	4/4 1/1	4/4 1/1
**NaOH**	CNS *S. aureus*	4/4 1/1	4/4 1/1	4/4 1/1	4/4 1/1

^1^n/N, where n is the number of isolates significantly inhibited by the indicated supernatant (p < 0.05) and N the number of isolates tested. For each isolate, the percentage of the remaining biofilm relative to a control biofilm without CNS supernatant was calculated based on the mean of 3 replicates. Statistical significance for the effect on biofilm formation was established for p-values less than 0.05 (p < 0.05).

## Discussion

Bacteria present in biofilms pose a serious risk to human and animal health [2,17,38,39]. This is particularly true for staphylococci, which are involved in many biofilm-associated infections, including infections related to medical device in human medicine and bovine mastitis in veterinary medicine [2,40]. Consequently, several studies have focused on developing new strategies against biofilm-forming bacteria, particularly *Staphylococcus* spp. [22,41,42]. A thorough understanding of the components and various stages of staphylococcal biofilm formation has enabled the development of alternative strategies [42]: (1) inhibiting the adhesion of bacteria in the planktonic form to a surface to prevent biofilm formation; (2) disrupting the biofilm architecture during the maturation phase; (3) inhibiting intra- and intercellular signals. Although these molecules were initially considered for use in human medicine, an increasing number of studies also highlight the importance of their use in veterinary medicine [22,43]. Furthermore, recent studies highlight alternative strategies such as those using compounds or extracts of microbial origin [22]. For example, outer membrane vesicles (OMVs) produced by *Pseudomonas aeruginosa* have been proposed as a strategy to inhibit the biofilm produced by *Streptococcus mutans* [44].

The results obtained by our group using mixed bacterial biofilms showed that four CNS bovine isolates exhibiting a weak-biofilm phenotype can significantly reduce biofilm formation and promote the dispersal of biofilm formed by staphylococci associated with bovine mastitis [4]. More recently, results have demonstrated the ability of *S. chromogenes* supernatants to reduce intramammary colonization by *S. aureus* [23]. Consequently, the objective of this study was to investigate and characterize the antibiofilm molecules produced by these four CNS isolates.

We first observed that the culture supernatants of the four CNS isolates (*S. chromogenes* C and E and *S. simulans* F and H) also had the ability to significantly impact the formation of staphylococcal biofilms, including a MRSA isolate (Fig 2–4), and these results are consistent with those of our previous study on mixed bacterial biofilms [4]. We observed an inhibitory effect on biofilm formation, but a much smaller effect was noted on the dispersion of pre-established biofilms (Fig 2 and 4). Furthermore, no effect on bacterial adhesion to surfaces was observed (S4 Fig). Additionally, the inhibition of biofilm formation does not appear to be due to inhibition of bacterial growth (S5 Fig), which is consistent with our previous study [4]. This type of antibiofilm property can be considered as an advantageous characteristic, as bactericidal activity could act as a selective pressure favoring bacterial adaptation and the emergence of resistance [45].

Our results also showed that the four supernatants exhibit a broad spectrum of activity that is not limited to other staphylococci (Tables 2 and 3). Indeed, we observed an inhibitory effect on biofilm formation by other Gram-positive bacteria (*S. uberis*, *S. dysgalactiae* and *T. pyogenes*), as well as an effect on the dispersion of pre-established biofilms produced by Gram-positive and Gram-negative bacteria (*K. oxytoca*, *K. pneumoniae* and *E. coli*) isolated from cases of bovine mastitis. This is also generally considered an advantage, as the therapeutic alternatives proposed to prevent or treat bovine mastitis, such as bacteriocins or bacteriophages, often have a narrow spectrum of activity [46,47]. Furthermore, multispecies biofilms are dominant in animal hosts and under most environmental conditions [48–51]. The supernatants produced by *S. simulans* isolates showed an even broader spectrum of activity than those produced by *S. chromogenes* isolates, suggesting that multiple molecules are most likely involved.

In this study, we also sought to gain further insight into the CNS-derived products present in the culture supernatants that are responsible for the antibiofilm activity. Characterization of the four supernatants indicated that the antibiofilm activity against most Gram-positive isolates was present in the < 3kDa fraction (Fig 6). Furthermore, the active molecules appear to be hydrophilic, heat-stable, and sensitive to RNase A (Tables 4 and 5). Results available at this time showed that antibiofilm activity was lost against 12 of the 22 CNS isolates after treatment of the supernatants with RNase A. Furthermore, preliminary results showed that antibiofilm activity was maintained with RNA extracts obtained from all four supernatants against a *S. chromogenes* isolate (S3 Fig). Enzymatic treatments also showed that antibiofilm activity was not affected by treatment with proteinase K. These results, combined with the absence of bactericidal activity, demonstrate that these CNS isolates do not appear to produce bacteriocins with bactericidal effects under the conditions tested in this study. Indeed, it is known that certain CNS isolates from dairy cows produce bacteriocins that exhibit antibacterial activity against other pathogens responsible for mastitis [52–55]. Our results differ from those of the study by Isaac et al. 2017, which describes the ability of exoproducts from a commensal strain of *S. chromogenes* to influence biofilm formation by staphylococci associated with bovine mastitis. Their results report active protein compounds with a molecular weight above 5 kDa [43]. Our results also differ from the study of Leroy et al. 2020, in which a molecule larger than 30 kDa present in the supernatant of *S. xylosus* strains was capable of inhibiting *S. aureus* biofilm formation [56]. Furthermore, we also demonstrated that alkalinisation of the active fraction has no effect on antibiofilm activity, suggesting that a quorum-sensing-like autoinducing peptide (AIP) is not involved [43]. Indeed, such an AIP, which could modulate agr quorum sensing activities with an impact on biofilm formation and dispersion in *S. aureus*, is labile at high pH and should be destroyed after treatment with NaOH. In contrast, we observed that the supernatants were not active against *S. aureus* after treatment with dispersin B. This difference could be explained by the fact that the composition of the extracellular matrix differs between CNS and *S. aureus*. Indeed, poly-beta (1,6)-N-acetyl-d-glucosamine (PNAG) the target of dispersin B, is not a major component of the CNS biofilm matrix, unlike in *S. aureus* [15,57].

In conclusion, this study demonstrated the antibiofilm activity of the four supernatants produced by CNS against several major pathogens causing bovine mastitis. This activity appears to be due, at least in part, to extracellular RNA (eRNA) molecules, which, to our knowledge, represents a mechanism that has never been described in the literature for CNS. There are reports indicating that eRNA contributes to the structural integrity of *Pseudomonas aeruginosa* and *S. aureus* biofilms [58,59]. Also, an increasing number of studies have highlighted the endogenous effect of small RNAs (sRNAs) on biofilm formation [60–63]. For example, overexpression of the sRNA RprA by *E. coli* reduces *csgD* expression and, consequently, biofilm formation [64]. The non-coding sRNA RsaE influences the composition of the extracellular matrix in *S. epidermidis* biofilm [65]. More recently, it has been demonstrated that the regulatory sRNA *teg58* plays an important role in modulating biofilm formation by *S. aureus* [66]. A trans-acting sRNA, SaaS, inhibits biofilm formation by *Salmonella* Enteritidis by interacting with the target mRNA [67].

However, very few studies had focused on extracellular sRNAs and their inter-species effects. Our initial list of RNA candidates is provided in S3 and S4 Tables and represents RNA found in either supernatants C and E from S. chromogenes or in supernatants F and H from S. simulans, respectively. Much more work will be needed to determine the ability of such RNA molecules to influence biofilm formation of other species. More generally, it has been proposed that the transfer of sRNAs produced by bacteria may be mediated by OMVs or extracellular vesicles and may modulate the physiology of neighboring bacterial cells, including biofilm formation, as well as the biological properties of host cells [68–70]. Given the novel nature of the antibiofilm fraction described here, its impact on staphylococci isolated in human medicine, as well as in multispecies biofilms, should also be evaluated in future studies. Indeed, these CNS supernatants could be explored as new antibiofilm strategies in veterinary or human medicine, either alone or in combination with antibiotics. However, additional studies should be conducted using *in vivo* methods to confirm these results. Indeed, microtiter plates are frequently used to rapidly screen the ability of microbes to form biofilms. Unfortunately, these are closed systems that are very different from environmental conditions.

## Supporting information

S1 Table Biofilm-inhibitory activity of the 4 supernatants from isolates of *S. chromogenes* and *S. simulans* exhibiting a weak biofilm phenotype (top) against five isolates exhibiting a strong biofilm phenotype, representing six species of staphylococci: *S. chromogenes*, *S. simulans*, S. *xylosus*, *S. epidermidis*, *S. haemolyticus* and *S. aureus.* Isolates numbers and symbols used in all Figures and Tables are indicated. A one-way ANOVA analysis was used to obtain the statistical data. * p-value less than 0.05 (p < 0.05); ** p < 0.01; *** p < 0.001. All experiments were performed on three independent days.
(PDF)

S2 Table Activity of the 4 supernatants from isolates of *S. chromogenes* and S*. simulans* exhibiting a weak biofilm phenotype (top) against pre-established biofilms of five isolates exhibiting a strong biofilm phenotype representing six species of staphylococci: *S. chromogenes*, *S. simulans*, *S. xylosus*, *S. epidermidis*, *S. haemolyticus* and *S. aureus.* Isolates numbers and symbols used in all Figures and Tables are indicated. A one-way ANOVA analysis was used to obtain the statistical data. * p-value less than 0.05 (p < 0.05); ** p < 0.01; *** p < 0.001. All experiments were performed on three independent days.
(PDF)

S3 Table Results of RNA sequencing. The list represents the overlap of RNA sequences found in both supernatants C and E from S. chromogenes.
(PDF)

S4 Table Results of RNA sequencing. The list represents the overlap of RNA sequences found in both supernatants F and H from S. simulans.
(PDF)

S1 Fig Electropherogram of RNA from BHI and C supernatants. Abbreviation: BHI, Brain Heart Infusion.
(TIF)

S2 Fig Electropherogram of RNA from E, F and H supernatants. (TIF)

S3 Fig Activity of RNA extracted from the supernatants of *S. chromogenes* and *S. simulans* on biofilm formation by an isolate of S. chromogenes (#102) exhibiting a strong biofilm phenotype. Activity of RNA extracted (SN PCl) from the supernatants of *S. chromogenes* (Sn C and Sn E) isolates and *S. simulans* (Sn F and Sn H) isolates exhibiting a weak biofilm phenotype (x axis) on the biofilm formation of *S. chromogenes* (#102). Biofilm formation is expressed as OD measured at 590 nm. A one-way ANOVA analysis was used to obtain the statistical data. * p-value less than 0.05 (p < 0.05); ** p < 0.01; *** p < 0.001. All experiments were performed on three independent days.
(TIF)

S4 Fig Anti-adhesive activity on surfaces of the 4 supernatants from isolates of *S. chromogenes* and S*. simulans* exhibiting a weak biofilm phenotype (in grey), as well as the BHI supernatant (in dark), against one isolate exhibiting a strong biofilm phenotype, representing six species of staphylococci: *S. chromogenes*, *S. simulans*, *S. xylosus*, *S. epidermidis*, *S. haemolyticus* and *S. aureus.* The effects of the supernatants on adhesion were calculated with the following formula = (100%) – (% of remaining biofilm relative to control), where 100% is the amount of biofilm formed in BHI+glucose, and were expressed as a percentage (%) of the remaining biofilm relative to a control biofilm without CNS supernatant. A one-way ANOVA analysis was used to obtain the statistical data. No statistical difference was observed. All experiments were performed on three independent days.
(TIF)

S5 Fig Effect of CNS supernatants (Sn C in pink, Sn E in red, Sn F in light purple, Sn H in dark purple) on the bacterial growth of *S. chromogenes*, *S. simulans*, *S. xylosus*, *S. epidermidis*, *S. haemolyticus* and *S. aureus.* Supernatant of BHI + glucose (in green) and water (in blue) were used as control.
(TIF)
