# Supplementary material for: Characterization of Antibiofilm Molecules from Bovine Coagulase-Negative Staphylococci
Source: PLoS One. 2026 Jun 30;21(6):e0351675. doi: 10.1371/journal.pone.0351675 (PMC13318025; doi:10.1371/journal.pone.0351675)
Supplement: S1 Table — Isolates numbers and symbols used in all Figures and Tables are indicated. A one-way ANOVA analysis was used to obtain the statistical data. * p-value less than 0.05 (p < 0.05); ** p < 0.01; *** p < 0.001. All experiments were performed on three independent days. (PDF) [file pone.0351675.s001.pdf]

Biofilm formation			Supernatants produced by isolates with a low ability to form a biofilm			
Isolates with a strong ability to form a biofilm	Species/strains		*S. chromogenes*		*S. simulans*	
			1180-1665 (C)	4021-4986 (E)	3140-6932 (F)	3100-0949 (H)
	Staphylococcus chromogenes	4080-0059 (101) ●	**	*	***	***
		4070-0281 (102) ■	*		*	*
		3200-1501 (103) ▲	***	***	***	***
		4120-6461 (104) ◆	***	***	***	***
		2250-0991 (105) ▼	*	**	***	***
	S. simulans	3230-0284 (106) ●			*	
		4170-4486 (107) ■				
		4080-7423 (108) ▲	***	***	***	***
		3130-0360 (109) ◆		**	**	***
		3230-1199 (110) ▼				
	S. xylosus	4120-6492 (111) ●	**	**	*	**
		4010-1170 (112) ■	***	***	***	***
		4180-2182 (113) ▲	***	***	***	***
		3130-0193 (114) ◆	***	**	**	*
		3191-5046 (115) ▼	***	***	***	***
	S. epidermidis	3210-4837 (116) ●	***	***	***	***
		2201-0445 (117) ■	**	*		*
		2141-3728 (118) ▲	***	***	***	***
		3210-0389 (119) ◆	***	***	***	***
		3210-1836 (120) ▼	***	**	***	***
	S. haemolyticus	4080-1063 (121) ●	***	***	***	***
		3200-1181 (122) ■	**	***		
		3260-1329 (123) ▲	***	***	***	***
		2170-4321 (124) ◆	***	***	***	***
		3030-8428 (125) ▼	***	***	***	***
	S. aureus	1061-3160 (126) ●	***	***	***	***
		2061-2859 (127) ■	***	***	***	***
		1070-5001 (128) ▲			*	*
		1020-0186 (129) ◆		**	***	***
		1081-2464 (130) ▼ (MRSA)	***	***	***	***
