# Supplementary material for: Characterization of Antibiofilm Molecules from Bovine Coagulase-Negative Staphylococci
Source: PLoS One. 2026 Jun 30;21(6):e0351675. doi: 10.1371/journal.pone.0351675 (PMC13318025; doi:10.1371/journal.pone.0351675)
Supplement: S3 Table — The list represents the overlap of RNA sequences found in both supernatants C and E from S. chromogenes. (PDF) [file pone.0351675.s003.pdf]

Gene Name	Start position	End position	Strand	Length	Old Gene Name	Gene ID	Description
DWB92_RS00255	55497	55931	+	435			Rrf2 family transcriptional regulator
DWB92_RS00260	55918	57240	+	1323			FAD-dependent oxidoreductase
DWB92_RS00305	64324	65621	+	1298			ISL3 family transposase
DWB92_RS00665	143846	145021	+	1176	deoB		phosphopentomutase
DWB92_RS00870	187160	187231	-	72			FAD-dependent oxidoreductase
DWB92_RS00920	208562	209785	+	1224			accessory Sec system protein translocase subunit SecY2
DWB92_RS00930	211342	212900	+	1559	asp2		accessory Sec system protein Asp2
DWB92_RS01230	285949	287343	+	1395			MFS transporter
DWB92_RS01905	430605	431891	+	1287			YSIRK-type signal peptide-containing protein
DWB92_RS01990	456103	456513	+	411			hypothetical protein
DWB92_RS01995	456461	456619	+	159			hypothetical protein
DWB92_RS02010	460284	466571	+	6288			YSIRK-type signal peptide-containing protein
DWB92_RS02105	487807	489000	+	1194			MFS transporter
DWB92_RS02365	548772	550010	+	1239			copper resistance protein CopC
DWB92_RS02465	571078	571626	+	549			TetR/AcrR family transcriptional regulator
DWB92_RS04670	969770	970147	+	378			LPXTG cell wall anchor domain-containing protein
DWB92_RS05155	1040934	1041395	+	462			hypothetical protein
DWB92_RS06315	1282103	1283542	+	1440			rhomboid family intramembrane serine protease
DWB92_RS08605	1769963	1770520	-	558			GNAT family N-acetyltransferase
DWB92_RS09055	1848980	1849570	+	591			CYTH domain-containing protein
DWB92_RS09360	1914408	1914863	+	456			hypothetical protein
DWB92_RS09680	1963682	1964920	-	1239			AAA family ATPase
DWB92_RS09750	1972166	1973214	+	1049			tyrosine-type recombinase/integrase
DWB92_RS10155	2047309	2047881	-	573			EMYY motif lipoprotein
DWB92_RS10160	2048034	2048552	+	519			hypothetical protein
DWB92_RS11570	2329614	2330525	-	912			cysteine synthase family protein
DWB92_RS11640	2343801	2344121	-	321			multidrug resistance protein SMR
