# Supplementary material for: Characterization of Antibiofilm Molecules from Bovine Coagulase-Negative Staphylococci
Source: PLoS One. 2026 Jun 30;21(6):e0351675. doi: 10.1371/journal.pone.0351675 (PMC13318025; doi:10.1371/journal.pone.0351675)
Supplement: S4 Table — The list represents the overlap of RNA sequences found in both supernatants F and H from S. simulans. (PDF) [file pone.0351675.s004.pdf]

Gene Name	Start position	End position	Strand	Length	Old Gene Name	Gene ID	Description
AL483_RS12770	16770	17969	-	1200		GeneID:32049513	GTP-binding protein
AL483_RS20170	1523690	1524322	-	633		GeneID:32050980	pathogenicity island protein
AL483_RS20175	1525005	1526660	-	1656		GeneID:32050981	DUF927 domain-containing protein
AL483_RS20190	1527902	1528312	-	411		GeneID:32050984	hypothetical protein
AL483_RS20320	1547381	1548070	+	690		GeneID:32051010	DUF4352 domain-containing protein
AL483_RS20410	1565342	1565701	-	360		GeneID:32051028	hypothetical protein
AL483_RS20420	1566151	1567794	-	1644		GeneID:32051030	terminase large subunit
AL483_RS20440	1568874	1570106	-	1233		GeneID:32051034	phage major capsid protein
AL483_RS20445	1570108	1570674	-	567		GeneID:32051035	HK97 family phage prohead protease
AL483_RS20450	1570661	1571794	-	1134		GeneID:32051036	phage portal protein
AL483_RS23040	2091678	2093678	-	2001		GeneID:32051541	CocE/NonD family hydrolase
AL483_RS23065	2098633	2099873	-	1241		GeneID:32051545	betaine/proline/choline family ABC transporter ATP-binding protein
AL483_RS23075	2100976	2101644	+	669		GeneID:32051547	response regulator transcription factor
AL483_RS23255	2138884	2139324	+	441		GeneID:32051583	MarR family transcriptional regulator
AL483_RS23445	2175228	2176550	-	1323	gabT	GeneID:32051621	4-aminobutyrate--2-oxoglutarate transaminase
AL483_RS24030	2316723	2317976	-	1254		GeneID:32051738	2-oxo acid dehydrogenase subunit E2
AL483_RS24040	2319056	2320003	-	948		GeneID:32051740	thiamine pyrophosphate-dependent dehydrogenase E1 component subunit alpha
AL483_RS24045	2320027	2321379	-	1353	lpdA	GeneID:32051741	dihydrolipoyl dehydrogenase
AL483_RS24725	2503458	2504714	+	1257		GeneID:32051869	MFS transporter
AL483_RS24730	2504731	2505510	+	780		GeneID:32051870	glucose 1-dehydrogenase
